# CT-based evaluation of thoracic anatomy in AS vs AR patients undergoing MIAVR

**DOI:** 10.1007/s11748-025-02223-1

**Published:** 2025-11-21

**Authors:** Yuto Yasumoto, Yoshitsugu Nakamura, Kasumi Tamagawa, Yuka Higuma, Kusumi Niitsuma, Miho Kuroda, Satoshi Okugi, Yujiro Hayashi, Taisuke Nakayama, Yujiro ito

**Affiliations:** Department of Cardiovascular Surgery, Chiba-Nishi General Hospital, 107-1 Kanagasaku, Matsudo, Chiba Japan

**Keywords:** Minimally invasive cardiac surgery, Aortic valve replacement, Aortic stenosis, Aortic regurgitation

## Abstract

**Objective:**

In this study, the difference in anatomical variables was considered between Aortic stenosis (AS) and Aortic regurgitation (AR) groups by preoperative computed tomography (CT) in minimally invasive cardiac surgery for aortic valve replacement (MIAVR).

**Methods:**

Patients who underwent AVR between 2012 and 2021 at our center were retrospectively analyzed in two groups, AS and AR. The final 278 samples possessed detailed clinical information of the patients. The six items were measured in preoperative CT and compared in significant difference in number.

**Results:**

No significant differences were found in the patients’ characteristics between the AS and AR groups except for age, sex and body surface area. The number of younger and male patients was higher in the AR group than in the AS group (*P* <  0.01), including a larger body surface area (*P* <  0.01). The AR group had larger rightward laterality aorta and third ICS (AS vs AR − 3.9 ± 8.9 vs 0.6 ± 8.9 mm, *P* < 0.01; 14.2 ± 1.3 vs 15 ± 1.6 cm, *P* <  0.01, respectively).

**Conclusions:**

Preoperative CT revealed thoracic anatomical differences between AS and AR patients undergoing MIAVR. Although no direct correlation with cross-clamp time was observed except for AP distance in both AR and AS, surgeons should be aware that these anatomical features—particularly the rightward aorta and deeper and more caudally positioned AV in AR patients to make surgical decision making, surgical planning. CT-based evaluation is a valuable tool for guiding approach strategy and patient selection in MIAVR.

## Introduction

Conventional aortic valve replacement (AVR) is a safe and effective procedure; however, minimally invasive AVR (MIAVR) is increasingly performed worldwide due to its advantages, including shorter hospital and ICU stays, reduced blood loss, lower incidence of postoperative atrial fibrillation, shorter ventilation time, and reduced hospital costs [1, 2].

While MIAVR is widely adopted, it presents technical challenges due to the limited operative field. These difficulties become more pronounced when thoracic anatomy is unfavorable [3]. In particular, variations in the position of the aortic valve—along lateral or cranio-caudal planes—can complicate key steps such as aortotomy, cardioplegia delivery, and valve implantation [4], often leading to longer aortic cross-clamp (XCL) and cardiopulmonary bypass (CPB) times [3, 5], both known contributors to postoperative morbidity and mortality [6, 7].

To address this issue, previous studies have proposed anatomical criteria and emphasized the utility of preoperative computed tomography (CT) to predict technical difficulty and guide patient selection [2, 3, 5–7]. However, few investigations have focused specifically on the differences in thoracic anatomy between patients with aortic stenosis (AS) and those with aortic regurgitation (AR). This distinction is clinically relevant because the surgical approach may be more complex depending on the underlying pathology.

This study aimed to assess and compare preoperative CT-derived thoracic anatomical features in AS and AR patients undergoing MIAVR, and to explore their potential impact on surgical access. Operative and clinical data were reviewed primarily as secondary, supporting measures.

## Materials and methods

**Fig. 1:**
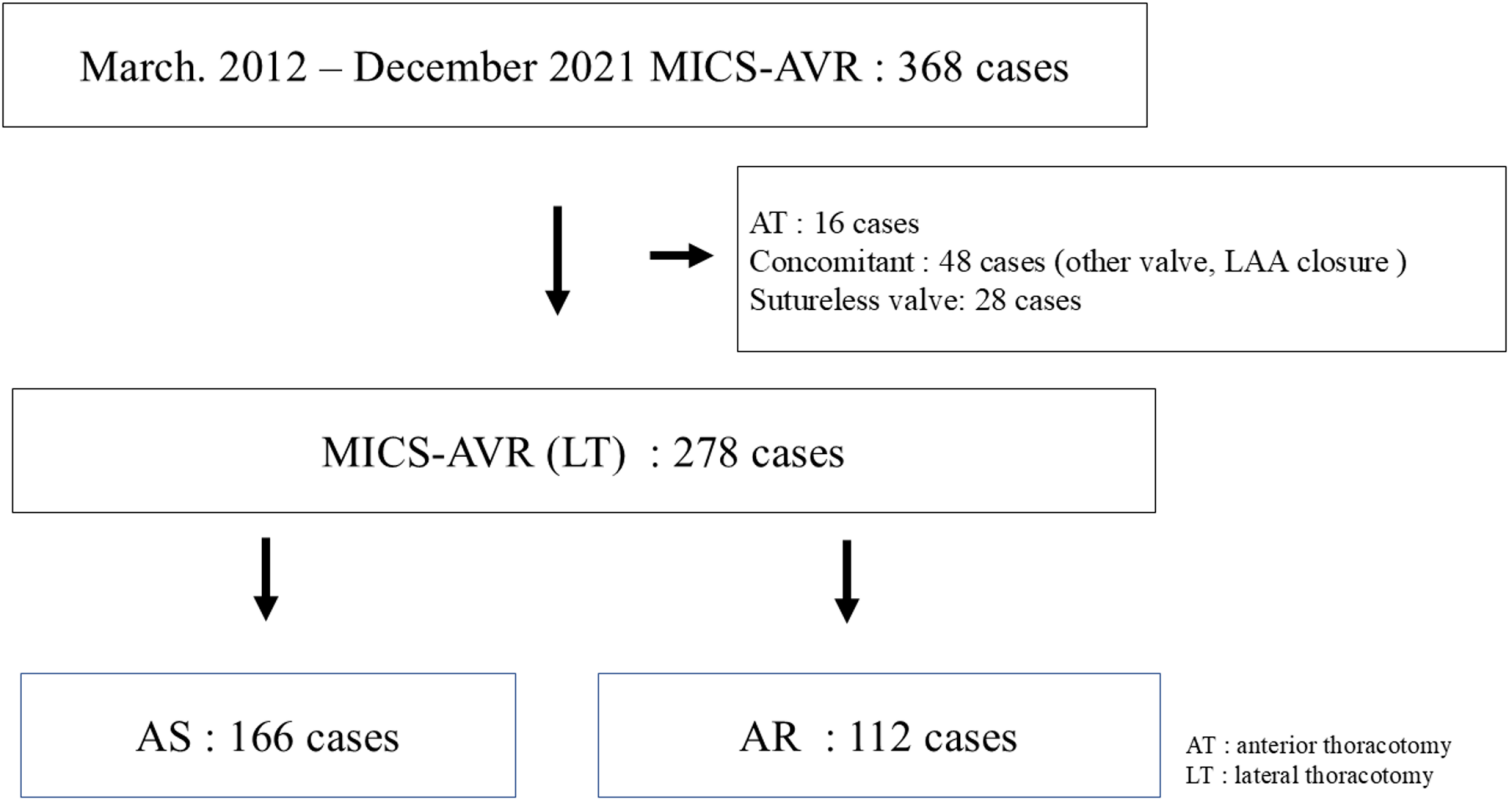
Patients selection and the cases for this report

This retrospective observational cohort study analyzed data from 368 consecutive patients who underwent AVR via a minimally invasive approach at Chiba-Nishi General Hospital between March 2012 and December 2021. After excluding cases involving right anterior thoracotomy (n = 16), concomitant procedures (n = 48), and sutureless valves (n = 28), 278 patients were included in the final analysis. Of these, 166 had AS and 112 had AR (Fig. 1). Institutional review board approval was obtained (IRB no. TGE02118-025), and the requirement for individual informed consent was waived. Preoperative CT was performed using at least 5 mm slice thickness with contrast enhancement in all patients. CT scans were performed with the both upper limbs elevated to maximize artifact reduction. However, at our hospital, CT scans for MIAVR are performed during exhalation to position the upper limbs downward and approximate the conditions of one-lung ventilation during artificial respiration.

## Surgical approach

All patients underwent AVR via a right lateral thoracotomy (LT), primarily chosen for high-risk or younger patients to avoid full sternotomy. Exclusion criteria for MIAVR included calcified ascending aorta, previous right lung surgery, poor left ventricular function (EF < 30–35%), or impaired pulmonary function (FEV1 < 1.0 L or interstitial pneumonia). Femoral artery cannulation was preferred, with axillary artery access selected in cases where retrograde perfusion was contraindicated due to severe vascular calcification or thrombus [8–10]. The patients in this study were part of a retrospective study; no cases involved changing the surgical plan based on CT findings. The procedure was performed through a 4–5 cm incision in the right third intercostal space (ICS), without sternotomy or cartilage resection. The surgery was performed under direct vision, with endoscopic assistance used as needed. Standard cardiopulmonary bypass and cardioplegia strategies were employed. Some cases took longer time due to factors such as distant surgical fields. Therefore, the aim of this study was to determine whether examining the anatomical relationships of the aorta and valves on CT could enable some prediction of these factors.

## CT-based anatomical measurements

**Fig. 2:**
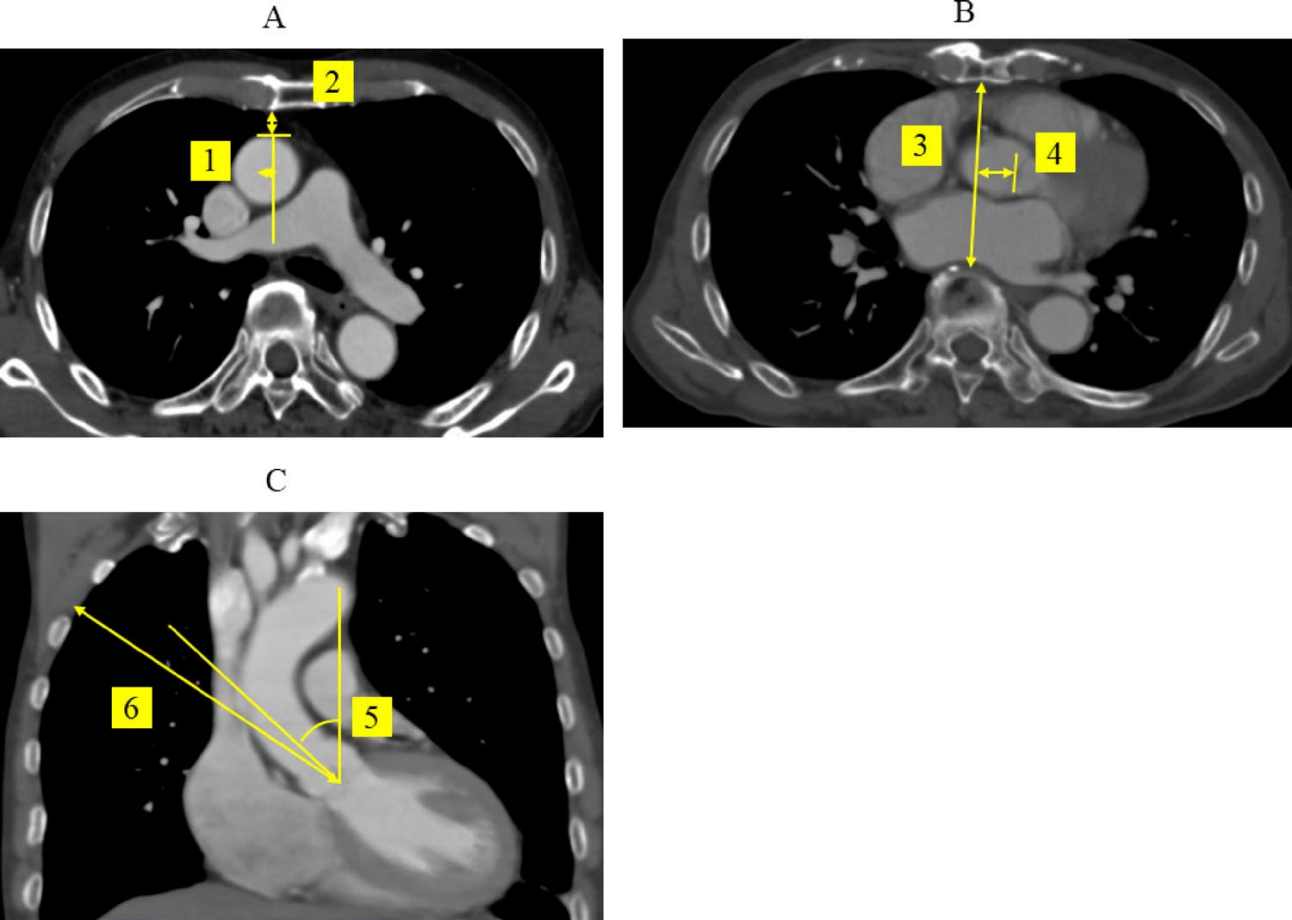
Preoperative CT: (1) laterality of aorta (dextroposition was measured as the distance of the aortic center lateral to the right sterna edge at the same level of the pulmonary trunk on axial image), (2) aorta depth (the distance from the posterior sternal edge to the anterior aortic edge at the level of the pulmonary trunk on axial image), (3) anteroposterior (AP) thoracic diameter (distance from the sternum to the vertebra at the level of the AV at the same level of AV on axial image), (4) AV deviation (distance from the median line to the AV at the same level of AV on axial image), (5) aortic angle (the angle between the ascending aorta and the vertical axis on the coronal image), and (6) the third ICS distance (the distance between the third ICS and the AV on the coronal image)

Preoperative CT images were reviewed and measured twice to minimize interobserver variability. The following six anatomical parameters were assessed (Fig. 2):

1. Laterality of the aorta—distance from the aortic center to the right sternal edge (axial view, at pulmonary trunk level)
2. Aortic depth—distance from posterior sternal edge to anterior aortic wall (the same level)
3. Anteroposterior (AP) thoracic diameter—from sternum to vertebral body at the aortic valve level
4. Aortic valve deviation—distance from midline to the aortic valve center
5. Aortic angle—angle between the ascending aorta and the vertical axis (coronal view)
6. Third ICS distance—vertical distance between the third ICS and the aortic valve (coronal view)
7. Distance to caudal of AV—calculated by distance from third ICS and angle of aorta

## Statistical analysis

Statistical analyses were performed using EZR (a modified version of R Commander). Continuous variables were compared using the Wilcoxon rank-sum test, and categorical variables using Fisher’s exact test or the χ2 test, as appropriate. Statistical significance was set at *P* < 0.05.

## Results

Table 1 presents detailed patient preoperative characteristics. The number of younger and male patients was higher in the AR group than in the AS group who also had a larger body surface area. No significant differences were found in the incidence of hyper pressure, chronic renal disease, chronic obstructive pulmonary disease, and risk scores between the AS and AR groups.

**Table 1:** Preoperative patients characteristics

	AS (N = 166)	AR (N = 112)	*P* value
Age (year-old)	74.9 ± 9.6	68.2 ± 12.5	< 0.01
Sex (female n, %)	92 (55%)	34 (31%)	< 0.01
Height (cm)	156.2 ± 9.0	162.2 ± 9.8	< 0.01
Weight (kg)	57.1 ± 11.0	59.8 ± 13.1	0.07
BSA (m^2)	1.52 ± 0.2	1.59 ± 0.2	< 0.01
euroSCORE Ⅱ (%)	2.3 ± 2.4	1.9 ± 2.7	0.24
*Comorbidities (n, %)*			
HT	117 (72)	83 (75)	0.42
DM	44 (26)	11 (10)	< 0.01
CKD	44 (26)	26 (23)	0.67
-on dialysis	14 (8)	1 (1)	< 0.01
COPD	9 (5)	4 (4)	0.57
Bicuspid valve (n, %)	12 (7.2)	3 (2.7)	0.6
*Transthoracic echocardiogram*			
pre ejection fraction (EF)	64.7 ± 10.6	61.2 ± 10.8	< 0.01
pre diastolic dimension (Dd)	50.1 ± 7.2	56.7 ± 8.0	< 0.01

BSA: body surface area, HT: hyper tension, DM: diabetic mellitus, CKD: chronic kidney disease, COPD: chronic obstructive pulmonary disease

Table 2 presents the comparison of thoracic anatomical features between AS and AR patients. No significant differences were observed in aortic depth, aortic angle, AP thoracic diameter, or aortic deviation from the midline.

**Table 2:** The comparison of thoracic anatomical features between AS and AR patients

	AS (Raw)	AR (Raw)	*P* value (Raw)	AS (Indexed)	AR (Indexed)	*P* value (Indexed)
1. Laterality of aorta (mm)	− 3.9 ± 8.9	− 0.6 ± 8.9	< 0.01	− 2.7 ± 6.0	− 0.5 ± 5.6	< 0.01
2. Depth of aorta (mm)	18.7 ± 7.4	17.9 ± 7.7	0.4	12.4 ± 4.9	11.3 ± 4.8	0.06
3. A-P distance (cm)	11.3 ± 1.9	10.9 ± 1.8	0.15	7.5 ± 1.6	6.9 ± 1.3	< 0.01
4. Deviation of AV (mm)	19.0 ± 7.7	17.2 ± 8.7	0.07	12.5 ± 5.4	11.0 ± 5.7	0.02
5. Angle of aorta (°)	43.0 ± 8.6	43.5 ± 10.1	0.62	31.3 ± 6.8	29.7 ± 7.4	0.05
6. Distance from third ICS (cm)	14.2 ± 1.3	15.0 ± 1.6	< 0.01	9.5 ± 1.4	9.6 ± 1.3	0.54
7. Distance to caudal of AV (cm)	9.6 ± 1.7	10.2 ± 2.1	0.01	6.4 ± 1.3	6.5 ± 1.5	0.50

“Raw” means the actually figures, “Indexed” means divided by body surface area for standardization

**Fig. 3:**
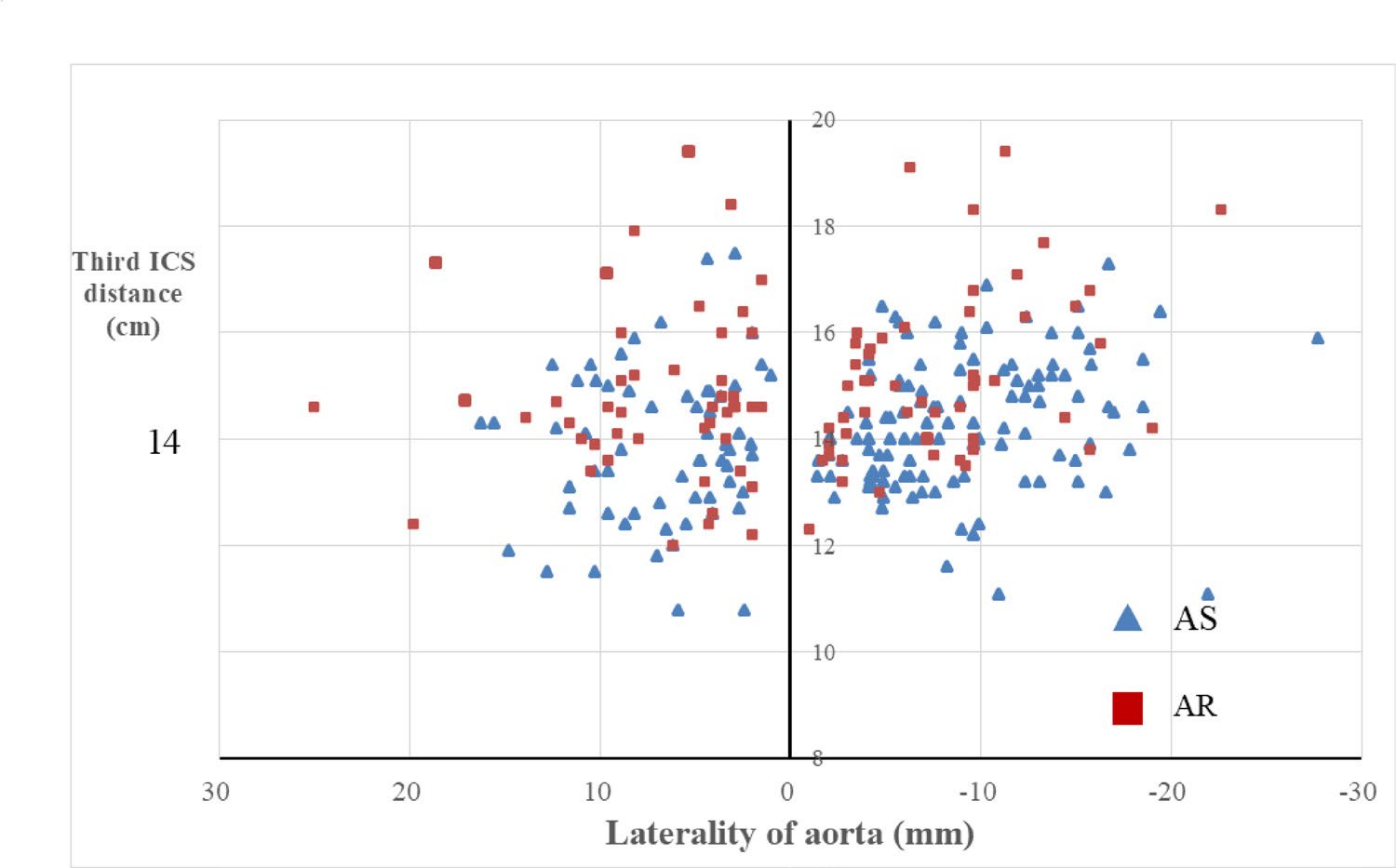
The chart showed preoperative CT measurement of patients with a larger laterality of the aorta and distance from the third ICS. Triangles showed AS patients, squares showed AR

**Fig. 4:**
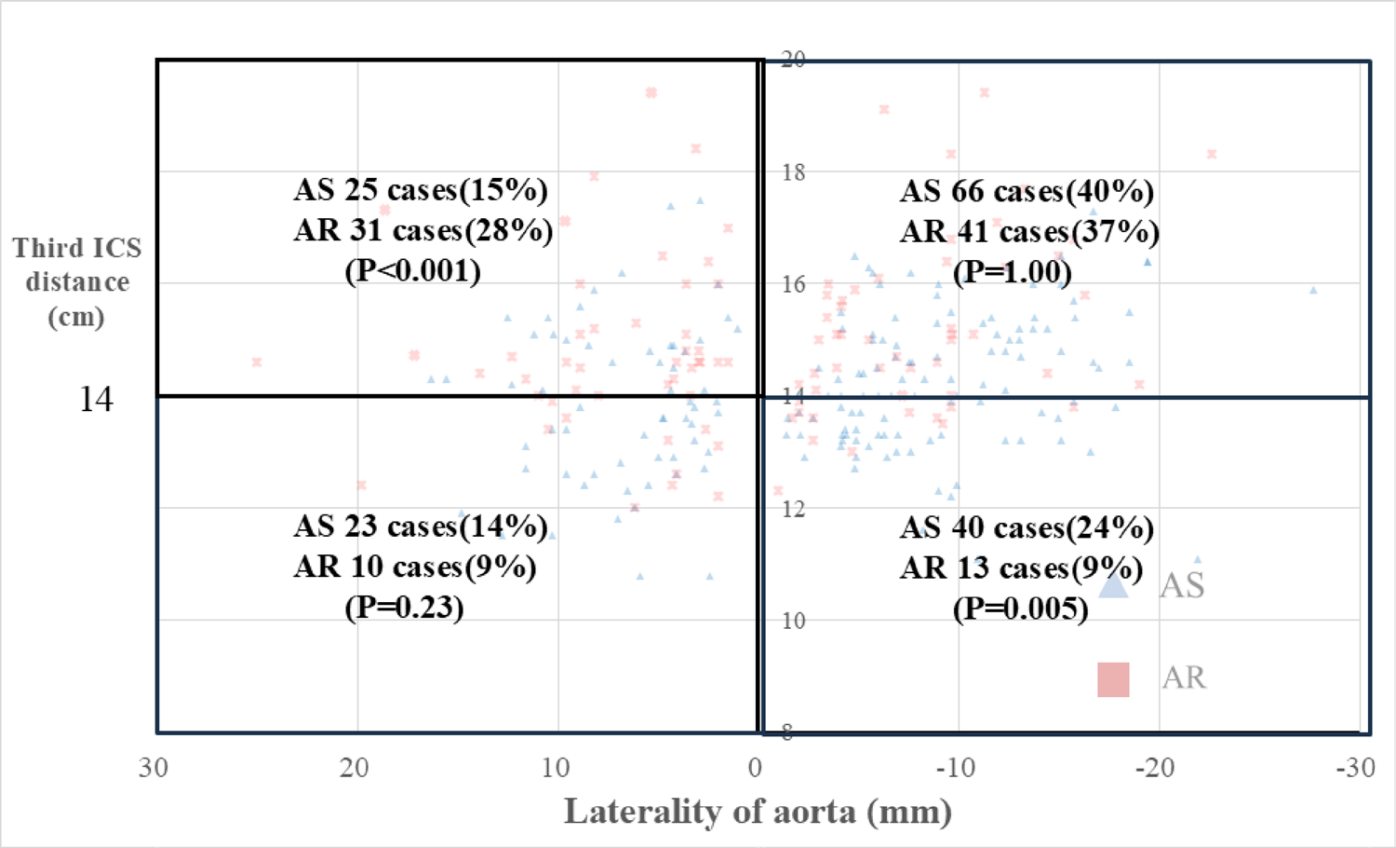
The chart showed preoperative CT measurement of patients with a larger laterality of the aorta and distance from the third ICS. The number of patients whose laterality was larger than 0 mm and the distance from third ICS was larger than 4 cm was higher in the AR group than in the AS group

However, the AR group had significantly greater laterality (AS: − 3.9 ± 8.9 mm vs. AR: 0.6 ± 8.9 mm, *P* < 0.01) and a longer third ICS distance (AS: 14.2 ± 1.3 cm vs. AR: 15.0 ± 1.6 cm, *P* < 0.01). These findings suggest that in AR patients, the aorta is more laterally positioned and located further from the third ICS, which may increase procedural complexity during MIAVR. When both variables were adjusted for BSA, only laterality remained significantly different (*P* < 0.05). Also, the AR group had significantly greater caudal distance of AV, when corrected by body surface area, there was no significant difference. Additionally, more AR patients had both marked aortic laterality and third ICS-to-aorta distance exceeding 14 cm (Figs. 3 and 4).

Figures 3 and 4 plot each patient based on the laterality of the aorta (horizontal axis) and the distance from the third ICS to the aortic valve (vertical axis), clearly showing differences between AR and AS patients.

 A subgroup with both aortic laterality greater than 0 mm and a third ICS-to-AV distance over 14 cm—features associated with higher surgical complexity—was identified. This subgroup included 25 patients (15%) with AS and 31 patients (28%) with AR, a significantly higher proportion in the AR group (*P* < 0.001).

Conversely, a subgroup with both aortic laterality smaller than 0 mm and a third ICS-to-AV distance under 14 cm was identified. This subgroup included 45 patients (24%) with AS and 13 patients (9%) with AR, showing a significantly higher proportion in the AS group (*P* <  0.005).

We also investigated the numerical difference due to sex, male group has larger size of AP distance and distance from third ICS to aortic valve, however, aortic valve laterality and deviation have no significant difference.

Table 3 showed the anatomical difference between the shorter and longer cross-clamp time in AS and AR. Both median cross-clamp time was 90.5 min, therefore, the group with a shorter time was designated as “short” and a longer time was “long”. The laterality of aorta and the distance from third intercostal space did not have significant difference. Overall, it was considered that extending the cross-clamp time might affect the AP distance and angle. However, no anatomically significant differences were observed in AS. In AR, it was suggested that the greater the AP distance, the longer the cross-clamp time might become.

**Table 3:** Multivariate analysis of the anatomical characteristics compared by cross-clamp time in AS and AR patients

	Odds ratio	VIF	*P* value
*Total*			
1. Laterality of aorta (mm)	0.98	1.64	0.43
2. Depth of aorta (mm)	1.01	1.64	0.61
3. A-P distance (cm)	1.25	1.54	0.02
4. Deviation of AV (mm)	1.06	1.94	0.31
5. Angle of aorta (°)	1.04	1.34	< 0.01
6. Distance from third ICS (cm)	0.86	1.29	0.12
*AS*			
1. Laterality of aorta (mm)	1.02	1.87	0.45
2. Depth of aorta (mm)	1.01	1.58	0.79
3. A-P distance (cm)	1.07	1.47	0.53
4. Deviation of AV (mm)	1.07	2.02	0.35
5. Angle of aorta (°)	1.04	1.40	0.12
6. Distance from third ICS (cm)	0.98	1.24	0.89
*AR*			
1. Laterality of aorta (mm)	0.96	1.57	0.22
2. Depth of aorta (mm)	1.02	1.68	0.67
3. A-P distance (cm)	1.61	1.59	< 0.01
4. Deviation of AV (mm)	1.05	2.21	0.21
5. Angle of aorta (°)	1.04	1.31	0.09
6. Distance from third ICS (cm)	0.99	1.49	0.96

The analysis indicated a tendency for the cross-clamp time to be prolonged when the odds ratio was greater than 1

We also examined the postoperative course, the breakdown of complications was as follows: symptomatic cerebral infarction in one case, wound infection in three cases, respiratory failure in three cases, acute renal failure in twenty-six cases, exploration in two cases, and heart failure in one case. Complications were observed in thirty-three cases overall. There were no cases of median conversion. Multivariate analysis of postoperative outcomes based on anatomical features was performed, the result was showed at Table 4. No significant difference was observed in postoperative complications due to anatomical differences on CT (Table 3).

**Table 4:** Multivariate analysis of postoperative complications based on anatomical features

Complications	Odds ratio	VIF	*P *value
1. Laterality of aorta (mm)	1.05	1.57	0.08
2. Depth of aorta (mm)	1.02	1.59	0.45
3. A-P distance (cm)	1.02	1.50	0.89
4. Deviation of AV (mm)	1.05	1.82	0.12
5. Angle of aorta (°)	1.02	1.31	0.32
6. Distance from third ICS (cm)	1.14	1.28	0.33

Complications include cerebral infarction, surgical site infection, respiratory failure, renal failure, exploration, or heart failure

## Operative time (supporting data)

For the entire cohort, mean XCL and CPB times were 94.8 ± 27.3 and 125.6 ± 33.5 min, respectively. Both durations were significantly shorter in the AR group than in the AS group (*P* < 0.01). However, no significant correlations were found between anatomical parameters and operative times, suggesting that surgical duration is influenced by multiple factors beyond thoracic anatomy.

## Discussion

From our result, the characteristics of AR patients compared to AS were a rightward displacement of the ascending aorta and the greater distance from the third intercostal space to the aortic valve, with a caudal displacement also present (Fig. 4, Table 2). As these are actual measurements, which may be influenced by body build and cardiac size. In reality, anatomy—including physical build—is a crucial factor. Multivariate analysis of CT results suggested that while they were not correlated with the incidence of postoperative complications.

This study highlighted distinct thoracic anatomical differences between AS and AR patients undergoing MIAVR, as assessed by preoperative CT. Notably, patients with AR have a more rightward the ascending aorta shifted to rightward instead the aortic valve did not follow rightward, which may complicate exposure during surgery. Although these features may not directly prolong operative times, they represent potential challenges, particularly for less experienced surgeons or early-phase programs. Importantly, MIAVR in this study was performed under direct vision, with endoscopic assistance used only for self-guidance. In fully endoscopic or robotic approaches, where the viewing axis and working space differ, the impact of anatomical factors may vary. Thus, the present findings should be interpreted within the context of direct-vision MIAVR. Aortas with laterality were predominantly right-sided in AR, and the distance from the third intercostal space to the AV was greater. However, converting this into an index negates the “Distance from third ICS” finding, making differences in body size a more likely explanation. Yet this no longer reflects the anatomical characteristics of AR patients. Deviation shows aortic valve of AS is more commonly left-sided, but Angle, while not significantly different at *P* =  0.05, tends to be steeper in AR, indicating AR is more caudally located. Whether this contributes to poor visual field requires further investigation.

Previous studies, including reports from Italy [3], have suggested that a rightward position of the ascending aorta is favorable for minimally invasive aortic valve replacement (MIAVR). These criteria were considered for MICS AVR via the AT approach and have implications on surgical procedures [11]. In general, a right-shifted ascending aorta is considered to facilitate surgical access and exposure. However, our analysis provides a more nuanced perspective. Figures 3 and 4 demonstrate that, although patients with AR tend to have a more rightward aorta compared with those with AS, they also often present with a greater distance from the third intercostal space to the aortic valve. From Table 2, the ascending aorta tends to be more rightward, and the aortic valve was shifted to more left side and deeper, and caudal. Corrected items eliminates that effect. This increased distance results in a longer and more oblique access pathway, which may limit maneuverability and visual exposure during MIAVR, potentially offsetting the anticipated advantage of a rightward aortic position. These findings highlight that it is not merely the position of the ascending aorta itself but rather the location of the aortic valve that determines the true technical difficulty. Relying solely on the rightward shift of the ascending aorta as a favorable criterion could lead to underestimating procedural complexity, particularly in AR patients. AR does not require time for leaflet resection, whereas AS requires time for calcification removal and may also require time for suturing calcified annuli. Furthermore, due to the prevalence of narrowed annuli, valve replacement requires careful planning and additional time. Careful preoperative imaging assessment that considers both laterality and depth is therefore essential to accurately predict surgical difficulty and guide patient selection for MIAVR.

This study’s main contribution lies in its detailed CT-based comparison of thoracic anatomy between AS and AR patients—an underexplored area in MIAVR research. While prior studies have proposed anatomical criteria for MICS-AVR, few have examined how valve pathology influences thoracic structure. Our findings support the use of preoperative CT not only to confirm feasibility but also to anticipate technical challenges specific to valve pathology.　Furthermore, prior studies have shown that patients with AR, particularly those with bicuspid valves, often have ascending aortic dilation or elongation [12–17], which can displace the valve caudally and complicate access. In the LT approach, no study has reported preoperative CT assessment in MIAVR, only MV repair [18]. Patients with AR were informed of the phenotypic modulation of smooth muscle cells in the ascending aorta, resulting in dilation and elongation [16]. The morphology of the AV affected the hemodynamics within the ascending aorta [19], which could lead to its elongation in patients with AR. These aortic changes may be less relevant in full sternotomy but can significantly impact MICS.

In the multivariate analysis of this study, thoracic anatomy showed no correlation with complications, but the influence of AP diameter on cross-clamp time was considered. While reports indicate that the transverse diameter of the thorax affects cross-clamp time in MICS mitral valve repair [20], the relationship between AP diameter and the procedure warrants further investigation.

In summary, although AR patients may appear anatomically favorable for MIAVR due to aortic dextroposition, the AV may be positioned deeper and more caudally, complicating exposure. CT-based anatomical assessment is therefore essential, especially in early-phase MIAVR.

## Limitations

1. This was a single-center retrospective study with a limited sample size, including patients with bicuspid valves. Multicenter validation studies are warranted to generalize the anatomical insights presented here.

2. How to handle body size correction. While correction might reveal true differences in thoracic anatomy between AS and AR, actual surgery prioritizes distance and anatomical positioning relative to body size. Therefore, this study uses actual measurements for discussion.

3. Patients with AR tend to have a larger left ventricular diameter compared to those with AS. Consequently, the heart may be relatively larger, and the aortic valve may be positioned more laterally, dorsally, or caudally. This study demonstrated that the aortic valve in AR patients is more caudally located; however, the relationship with LVDd, laterality, or dorsality remains unclear. It is difficult to make a determination based solely on disease-specific anatomical differences.

## Conclusion

Preoperative CT revealed thoracic anatomical differences between AS and AR patients undergoing MIAVR. Although no direct correlation with cross-clamp time was observed except for AP distance in both AR and AS, surgeons should be aware that these anatomical features—particularly the rightward aorta and deeper and more caudally positioned AV in AR patients to make surgical decision making, surgical planning. CT-based evaluation is a valuable tool for guiding approach strategy and patient selection in MIAVR.

## Abbreviations

AR: Aortic regurgitation
AS: Aortic stenosis
AT: Anterior thoracotomy
AV: Aortic valve
AVR: Aortic valve replacement
CT: Computed tomography
ICS: Intercostal space
LT: Lateral thoracotomy
MICS: Minimally invasive cardiac surgery
